# Attenuation of cancer-initiating cells stemness properties by abrogating S100A4 calcium binding ability in head and neck cancers

**DOI:** 10.18632/oncotarget.12935

**Published:** 2016-10-26

**Authors:** Li-Hao Cheng, Kai-Feng Hung, Tung-Fu Huang, Hsin-Pei Hsieh, Shu-Ying Wang, Chih-Yang Huang, Jeng-Fan Lo

**Affiliations:** ^1^ Institute of Oral Biology, National Yang-Ming University, Taipei, Taiwan; ^2^ Department of Dentistry, School of Dentistry, National Yang-Ming University, Taipei, Taiwan; ^3^ School of Medicine, National Yang-Ming University, Taipei, Taiwan; ^4^ Department of Orthopedics and Traumatology, Taipei Veterans General Hospital, Taipei, Taiwan; ^5^ Department of Microbiology and Immunology, Medical College, National Cheng Kung University, Tainan, Taiwan; ^6^ Graduate Institute of Chinese Medical Science and Institute of Medical Science, China Medical University, Taichung, Taiwan; ^7^ Institute of Basic Medical Science, China Medical University, Taichung, Taiwan; ^8^ Department of Health and Nutrition Biotechnology, Asia University, Taichung, Taiwan; ^9^ Department of Dentistry, Taipei Veterans General Hospital, Taipei, Taiwan; ^10^ Genome Research Center, National Yang-Ming University, Taipei, Taiwan; ^11^ National Yang-Ming University VGH Genome Research Center, Taipei, Taiwan

**Keywords:** epithelial-mesenchymal transition, head and neck squamous cell carcinomas, GRP78, p53, Nanog

## Abstract

S100A4 is a calcium-binding protein capable of promoting epithelial-mesenchymal transition. Previously, we have demonstrated that S100A4 is required to sustain the head and neck cancer-initiating cells (HN-CICs) subpopulation. In this study, to further investigate the molecular mechanism, we established the head and neck squamous cell carcinoma (HNSCC) cell lines stably expressing mutant S100A4 proteins with defective calcium-binding sites on either N-terminal (NM) or C-terminal (CM), or a deletion of the last 15 amino-acid residues (CD). We showed that the NM, CM and CD harboring sphere cells that were enriched with HN-CICs population exhibited impaired stemness and malignant properties *in vitro*, as well as reduced tumor growth ability *in vivo*. Mechanistically, we demonstrated that mutant S100A4 proteins decreased the promoter activity of Nanog, likely through inhibition of p53. Moreover, the biophysical analyses of purified recombinant mutant S100A4 proteins suggest that both NM and CM mutant S100A4 were very similar to the WT S100A4 with subtle difference on the secondary structure, and that the CD mutant protein displayed the unexpected monomeric form in the solution phase.

Taken together, our results suggest that both the calcium-binding ability and the C-terminal region of S100A4 are important for HN-CICs to sustain its stemness property and malignancy, and that the mechanism could be mediated by repressing p53 and subsequently activating the Nanog expression.

## INTRODUCTION

Head and neck squamous cell carcinoma (HNSCC) is a devastating cancer often refractory to chemotherapy and/or radiotherapy [1, 2]. The resistance of HNSCC to chemotherapy and/or radiotherapy is at least partly attributable to the subpopulation of cancer initiating cells (CICs), which exhibit stemness property and are capable of initiating carcinogenesis or promoting metastasis [3–9]. For this reason, targeting the CICs in HNSCC would be an appealing modality for the treatment of this type of cancer. However, the molecular mechanism by which cancer cells sustain the HN-CICs subpopulation is still unknown.

The epithelial-mesenchymal transition (EMT) is a process that loss of cell-cell adhesion and polarity are accompanied with increased cell motility via cytoskeleton rearrangements [10]. The EMT has also been implicated in the process for cells to acquire the CIC phenotype, thereby increasing the stemness property [11–14]. Furthermore, we also demonstrated that HN-CICs promote EMT and stemness properties by CD133/Src signaling [15].

S100A4, a calcium-binding protein, has been associated with the development of a metastatic phenotype [16–18]. Induction of EMT phenotype can be activated by Wnt/β-catenin signaling pathway. Previous studies show that S100A4 is a direct β-catenin/TCF target and highly associated with metastasis and poor survival in cancer patients [19–21]. In addition, S100A4-defiecient mice displayed less tumor formation and metastasis [22]. Further, presence of calcium has been demonstrated to be essential for S100A4 recognition of target proteins and the maintenance of S100A4 inducing properties [23–25]. In our previous study, we show that S100A4 acts as a stemness-maintaining factor for HN-CICs [26]. However, the regulation mediated by S100A4 and the downstream molecular targets to control HN-CICs stemness capability are remained unclear.

Previously, we have demonstrated that S100A4 play a crucial role in the maintenance of head and neck cancer-initiating cells (HN-CIC) population. In the current study, we found that abrogation of calcium-binding activity of S100A4 reduced the stemness properties of HN-CICs. In addition, S100A4 may maintain the stemness properties by diminishing the negative regulation of p53 on Nanog. Overall, our results suggest that the calcium-binding ability of S100A4 is important for self-renewal and stemness properties of HN-CICs.

## RESULTS

### Loss of stemness properties of HN-CICs by overexpressing mutant S100A4 proteins

**Figure 1:**
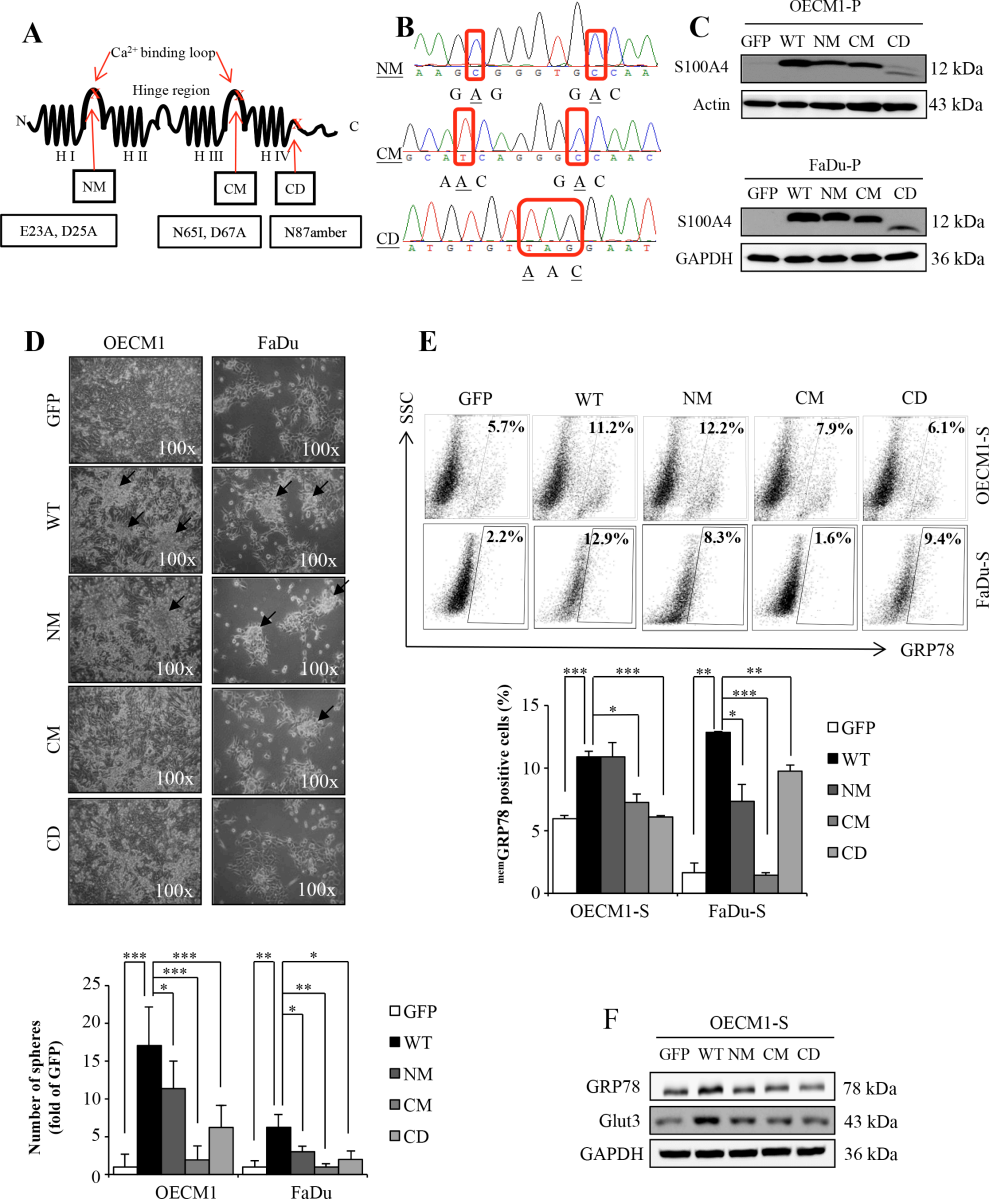
The mutants S100A4 attenuate the stemness properties of HN-CICs (**A**) The secondary structure and point mutation of S100A4. H: helices. (**B**) DNA sequencing data of NM (E23A, D25A), CM (N65I, D67A) and CD (N87amber). (**C**) Protein level of S100A4, Actin and GAPDH in OECM1-parental (P) and FaDu-parental (P) was determined by immunoblot analyses. (**D**) Representative images of sphere formation ability of GFP, WT, NM, CM and CD expressing OECM1 and FaDu cell lines. Black arrow indicated the sphere. (*p < 0.05; **p < 0.01; ***p < 0.001) (**E**) Cell surface GRP78 of GFP, WT, NM, CM and CD expressing sphere cells of OECM1 and FaDu were analyzed by flow cytometry (*p < 0.05; **p < 0.01; ***p < 0.001). (**F**) Protein level of GRP78, Glut3 and GAPDH in sphere cells of OECM1 by immunoblot analyses. Data are represented as mean ± SD.

Our previous study demonstrated that S100A4 is required to sustain the stemness and self-renewal property of the head and neck cancer-initiating cells (HN-CICs) population both *in vitro* and *in vivo* [26]. To further investigate how S100A4 is involved in stemness property, we established stable HNSCC cell lines expressing different dysfunctional S100A4, of which the calcium-binding site on N-terminal (NM) or C-terminal (CM) were mutated because loss of the calcium-binding activity of S100A4 abrogates its physiological function and interaction to the target proteins [24, 25]. In addition, we also generated cells expressing truncated S100A4 with the last 15 amino acids deleted (CD) (Figure 1A). Since these stable cell lines were generated via lentiviral vector co-expressing a green fluorescent protein (GFP), the GFP positive cells would harbor these different mutant S100A4, as verified by DNA sequencing (Figure 1B) as well as immunoblotting analysis (Figure 1C and Supplementary Figure S1C and S4B) using GFP-sorted cells. To examine the effect of these mutated S100A4 on the stemness property, we used sphere formation assay [27]. We found that cell harboring CM and CD S100A4 protein significantly reduced the sphere formation ability under the 2-week sphere forming cultivation (Figure 1D). Meanwhile, as we previously demonstrated that cells with expression of GRP78 on cell membrane (^mem^GRP78) or upregulation of the glucose transporter Glut3 exhibit high stemness property and enhanced *in vivo* tumorigenicity [9, 28], we then examined the levels of GRP78 and Glut3 in HN-CICs expressing mutant S100A4. We showed that the GRP78 positive population is reduced when cells express mutant S100A4 (Figure 1E). In addition, among the sphere HN-CICs harboring CM and CD mutant S100A4, the levels of GRP78 and Glut3 were reduced compared to cells with wild type S100A4 (Figure 1F). Collectively, these results suggest that enriched HN-CICs expressing mutants S100A4 exhibit reduced stemness property.

### Mutant S100A4 impairs the *in vivo* tumorigenicity of HNSCC cells

**Figure 2:**
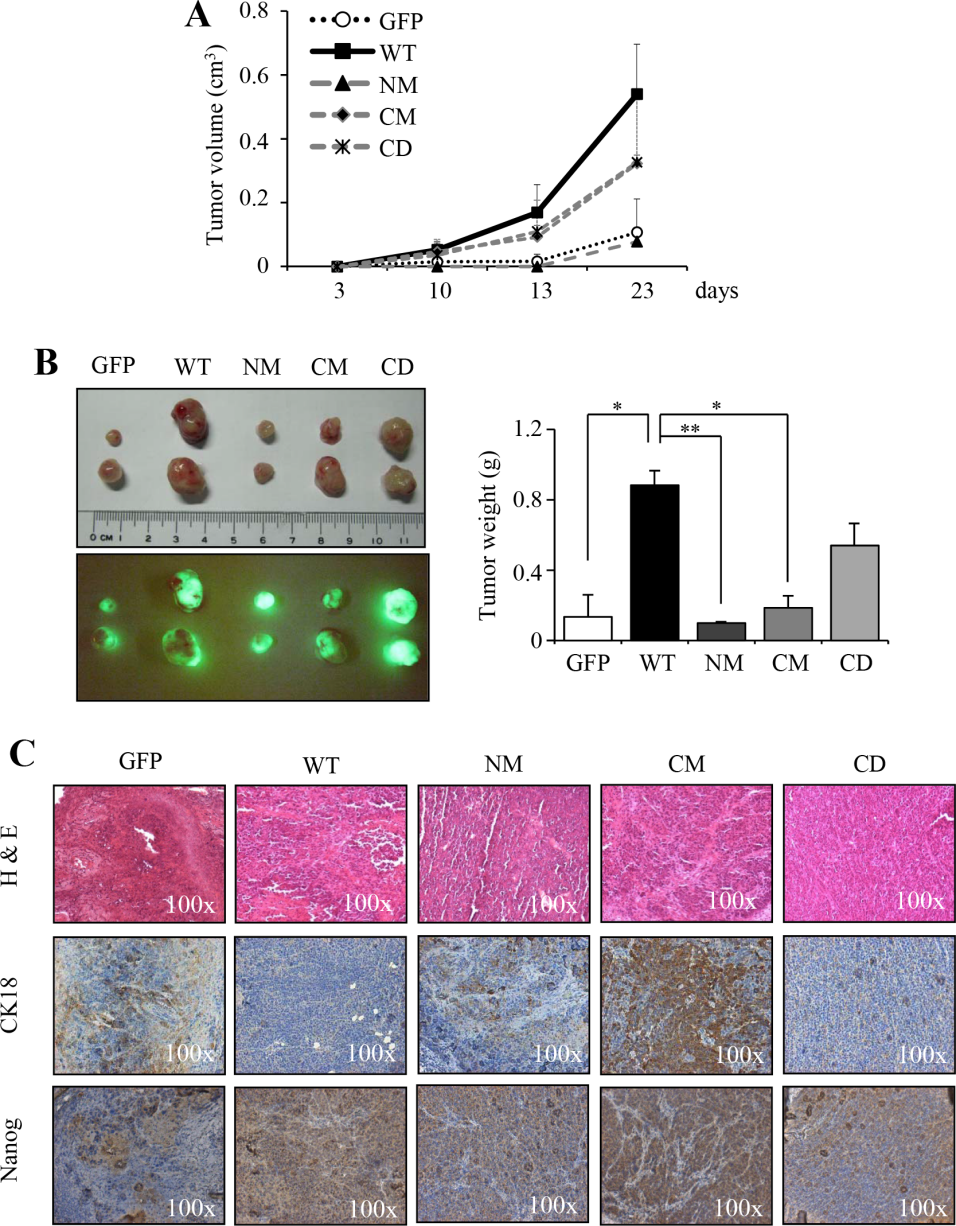
The mutants S100A4 impairs *in vivo* tumorigenic properties (**A**) FaDu-P (5 × 10^5^) were subcutaneously implanted into nude mice (*n* = 2). The tumor growth ability of GFP, WT, NM, CM and CD expressing cells were shown. (**B**) Image of dissected tumors was collected on day 23. The tumor weight was recorded (*p < 0.05; **p < 0.01). (**C**) The histopathologic analysis of tumor of GFP, WT, NM, CM, and CD by H&E-stained, respectively. The expression of Nanog and CK18 of tumors was examined by immunohistochemical analysis. Data are represented as mean ± SD.

We have shown that overexpression of S100A4 protein promotes the *in vivo* tumorigenicity of HNSCC [26]. To investigate whether overexpression of mutant S100A4 impairs the *in vivo* tumorigenicity of HNSCC, cells stably expressing wild type or mutant S100A4 (NM, CM and CD) were injected into nude mice. We observed that cells expressing mutant S100A4 are impaired in the tumor growth (Figure 2A) with reduced tumor weight (Figure 2B right panel), compared to the cell harboring WT S100A4. Notably, the tumors derived from HNSCC cells expressing WT S100A4 were shown with strong positivity of Nanog and weak CK18 immunostaining (Figure 2C). Collectively, these results suggest that the *in vivo* tumorigenicity is attenuated by the expression of mutant S100A4, thus promoting cells toward a more differentiated status in comparison to the cells expressing wild type S100A4.

### S100A4-mediated inhibition of p53 activates the expression of Nanog

**Figure 3:**
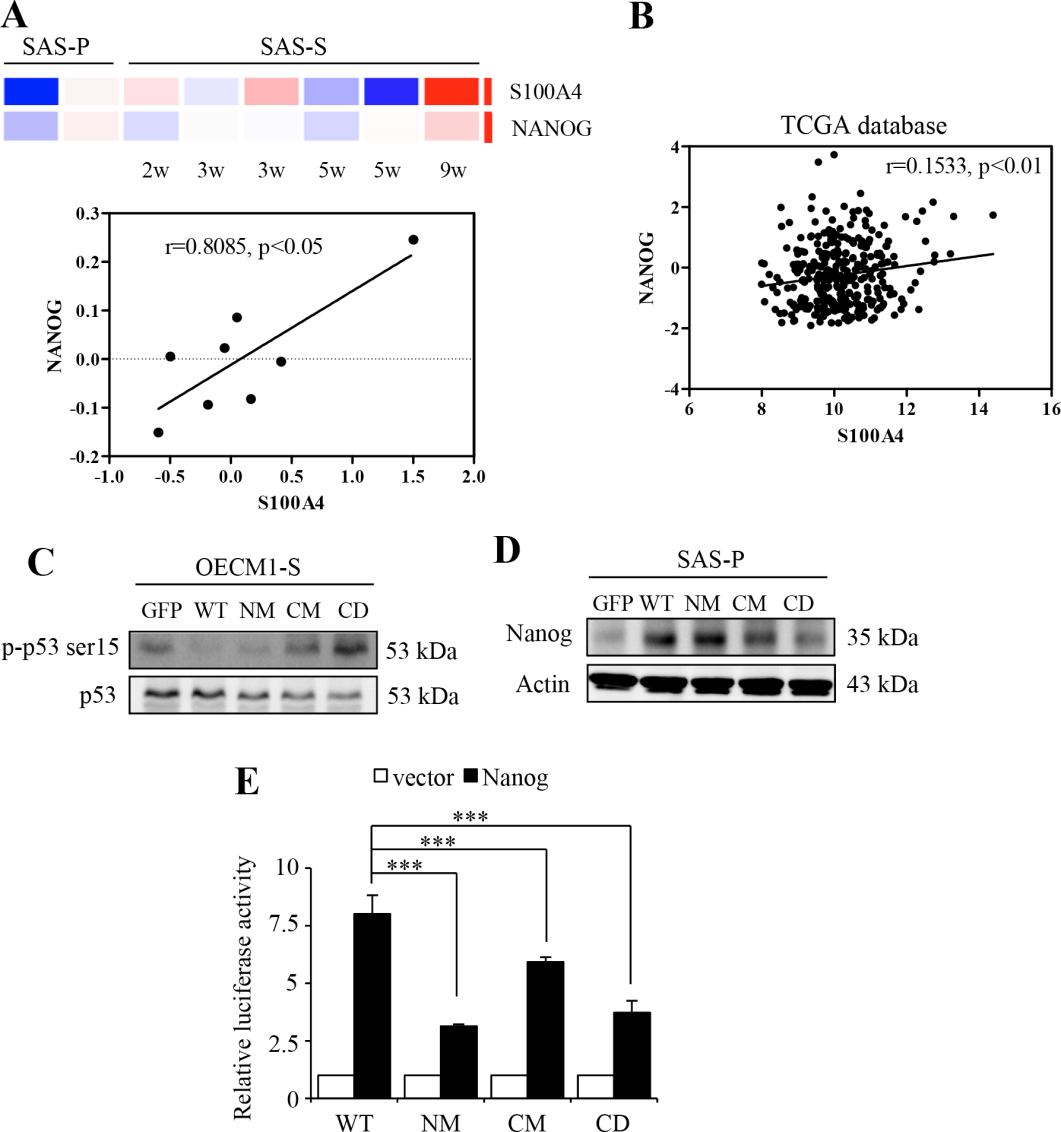
S100A4 enhances the expression of Nanog by negative regulating p53 (**A**) The heat maps of the S100A4 and Nanog of sphere cells. Red and blue determine as high and low expression levels, respectively (top panel). Correlation analysis between S100A4 expression (X-axis) and the expression of Nanog (Y-axis) (bottom panel) (**B**) Co-expression analysis for S100A4 in human head and neck cancer samples versus Nanog (*n* = 427). RNA-Seq plotted data are log_2_ mRNA expression from TCGA Research Network. (**C**) Protein level of p-p53 ser15 and p53 in OECM1-S by immunoblot analyses. (**D**) Protein level of Nanog and Actin in SAS-P by immunoblot analyses. (**E**) Representative relative luciferase activity of WT, NM, CM and CD of 293T cells compared to the promoterless vector was shown (***p < 0.001). Data are represented as mean ± SD.

It is essential that S100A4 proteins have to firstly bind with calcium, consequently, to exhibit the capability to recognize with the target proteins [24, 25, 29, 30]. Of the target proteins, tumor suppressor protein p53 is one of the direct targets of S100A4 [23, 31, 32]. Further, Lin et al. have reported that p53 induce differentiation of stem cells by suppressing the transcriptional activity of stem cell marker, Nanog [33]. To investigate the mechanism by which S100A4 promotes the stemness and tumorigenicity of HNSCC cells, we examine the level of Nanog and its association with p53. As shown in Figure 3A, we found that the S100A4 mRNA expression is significantly positive correlated with Nanog in HN-CICs sphere cells (*p* < 0.05). Importantly, using previously established xenograft-derived metastatic HNSCC cell lines [34, 35], we demonstrated that the expressions of S100A4 and, to less extend, the Nanog, are increased during the acquisition of metastatic phenotype (Supplementary Figure S2). Consistently, using The Cancer Genome Atlas (TCGA) database, we observed a statistically significant positive correlation between the expression profiles of S100A4 and Nanog transcripts in head and neck cancer (Figure 3B; *p* < 0.01).

Our prior study has found that S100A4 knockdown causes significant changes of TP53 by network topological analysis [26]. In addition, it was previously shown that S100A4 binds to the p53 that leads to inhibition of phosphorylation by protein kinase C (PKC) [32], and that S100A4 promotes the degradation of p53 in the nucleus [36]. Therefore, we hypothesize that calcium-bound S100A4 negatively regulates p53, leading to activation of Nanog transcriptional activity in HN cancers. Using phosphorylation of p53 at Ser15 (p-p53 Ser-15) as an indicator of p53 activation [37–42], we found that the level of p-p53 Ser-15 in HN-CICs sphere cells is attenuated upon expression of CM or CD S100A4 protein compared to sphere cells expressing WT S100A4 (Figure 3C). We also demonstrated that the level of Nanog is decreased in cells with expression of mutant S100A4 (Figure 3D). Moreover, to further elucidate whether the regulatory role of S100A4 in the expression of Nanog is mediated by p53 which directly binds to the promoter region of Nanog and down-regulates its transcription activity [33], we cloned a 1.2-kb genomic DNA containing the Nanog promoter for further reporter assay. We found that cells harboring mutants S100A4 exhibit significantly decreased reporter activity compared to the cells harboring WT S100A4 (Figure 3E). These results suggest that S100A4 sustains the stemness property of HNSCCs likely via the p53-mediated suppression of Nanog.

### S100A4 mutation abrogates the *in vitro* malignancy of HNSCC

**Figure 4:**
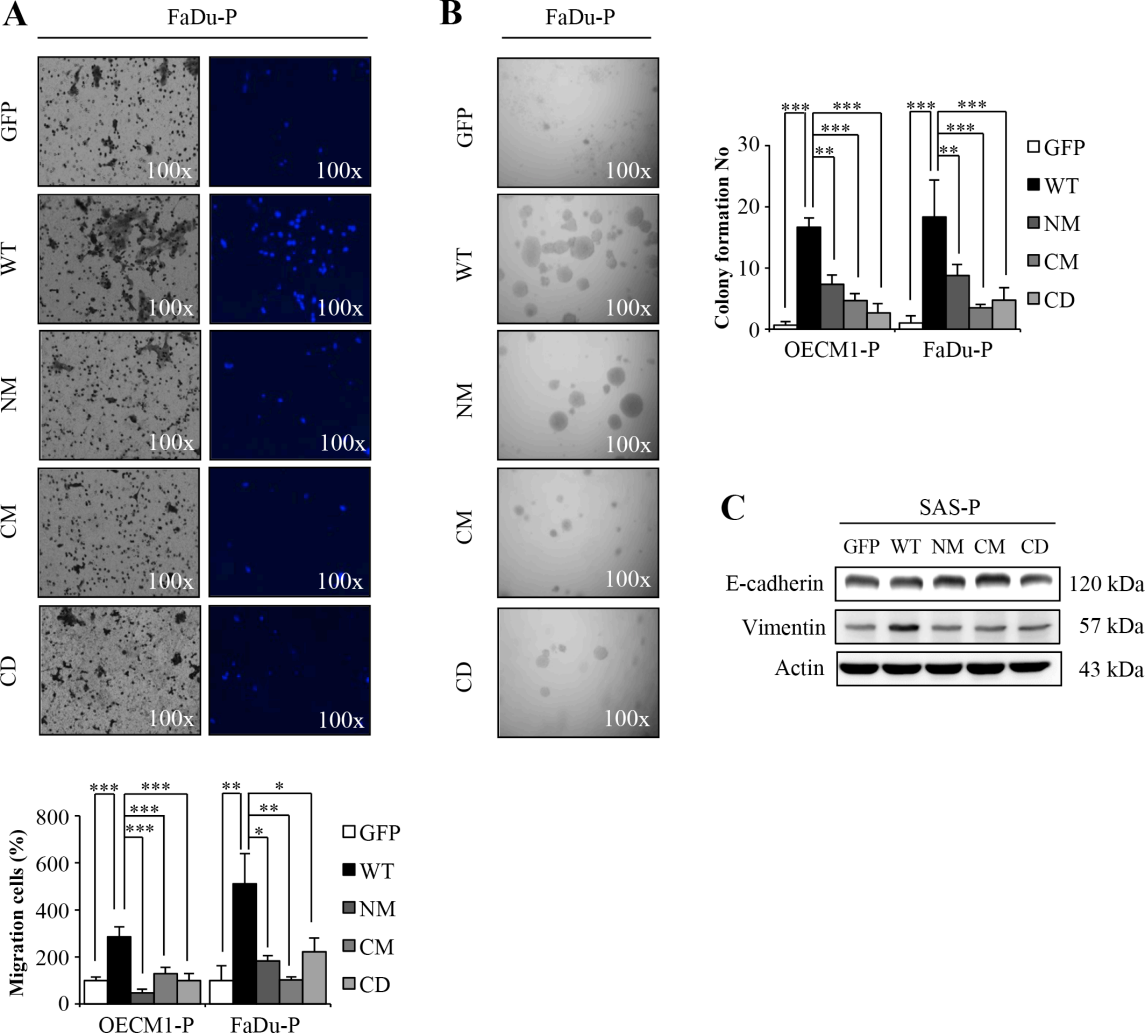
The mutants S100A4 decrease the tumorigenic capacity of HNSCC (**A**) The migration ability of OECM1-P and FaDu-P cell lines were examined as described in materials and methods (*p < 0.05; **p < 0.01; ***p < 0.001). (**B**) Anchorage-independent growth of S100A4-overexpressing OECM1-P and FaDu-P cell lines were analyzed (**p < 0.01; ***p < 0.001). (**C**) Protein level of E-cadherin, Vimentin and Actin in SAS-P by immunoblot analyses. Data are represented as mean ± SD.

To demonstrate the *in vitro* malignancy of cells harboring mutant or wild type S100A4 proteins, we examined the cell motility and anchorage independent growth ability of cells expressing NM, CM or CD mutant S100A4. Consistent with the idea that S100A4 promotes the malignancy; we showed that mutant S100A4 significantly decreased cell migration (Figure 4A and Supplementary Figure S3A) and anchorage independent growth (Figure 4B and Supplementary Figure S3B). In addition, we also observed that the epithelial marker E-cadherin was reduced and the mesenchymal marker Vimentin was increased in cells with mutants S100A4 (Figure 4C and Supplementary Figure S4A). Together, these data suggest that the *in vitro* tumorigenicity of HNSCC cells is abrogated by expression of mutant S100A4 defective in calcium-binding ability.

### Biophysical characterization of mutant S100A4

**Figure 5:**
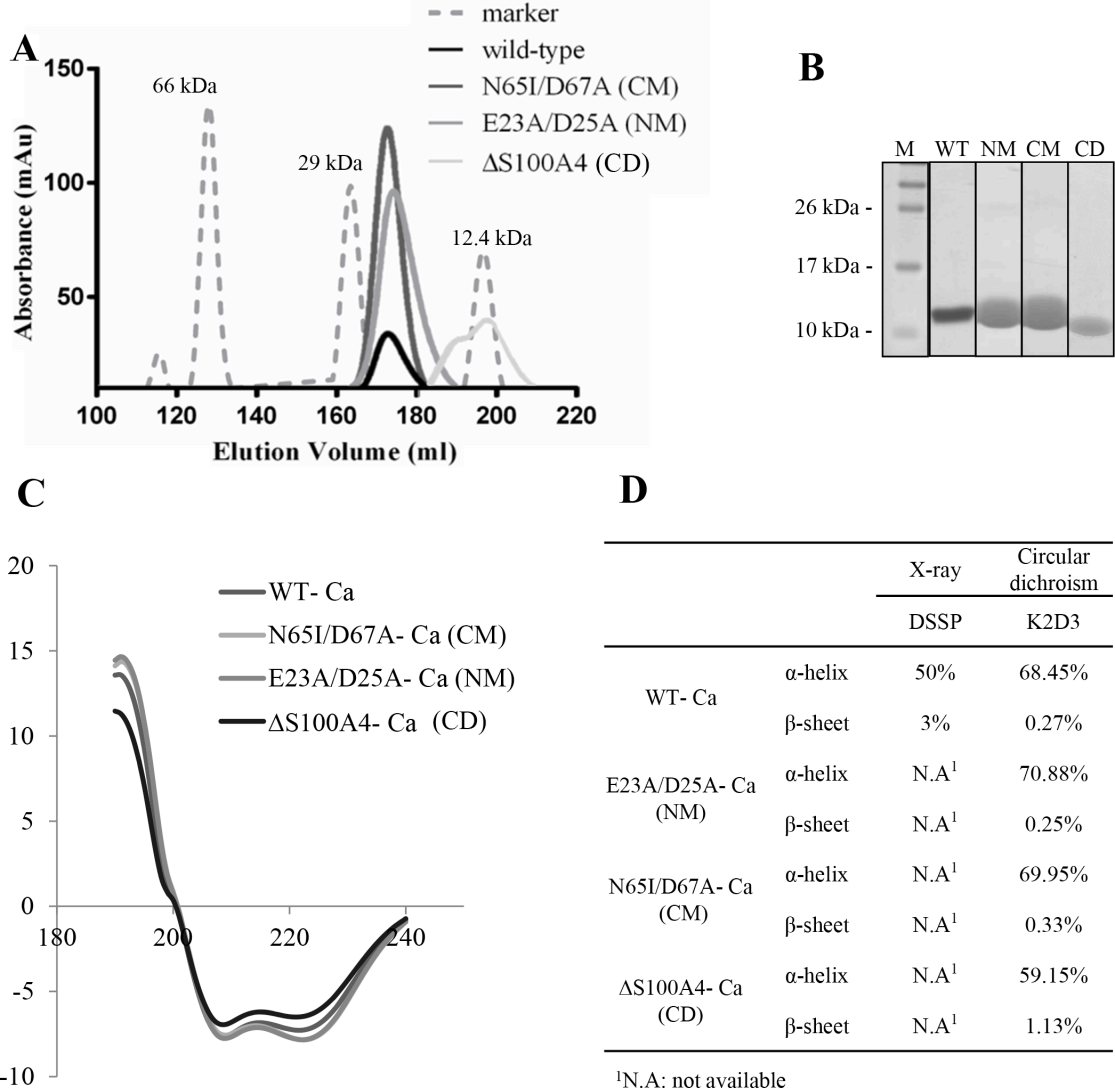
Biophysical characterization of S100A4 (**A**) Profiles of S100A4 WT and mutants for size-exclusion chromatography on HiLoad^TM^ 26/60 Superdex^TM^ 75 prep grade column. (**B**) SDS-PAGE of purified S100A4 proteins from size-exclusion chromatography. Molecular weight markers (M). (**C**) Circular dichroism measurements were determined in the amide band (190–240 nm) at 3 mg/ml protein concentration in S100A4 WT and mutants FPLC buffer using a Jasco-715 spectropolarimeter. The raw data were further analyzed by K2D3 software for production of smooth maps [50]. (**D**) X-ray and circular dichroism-calculated secondary structures for Ca^2+^-bound S100A4 WT and mutants.

To investigate the structural change and functional relationship of mutants S100A4, the recombinant proteins of three mutants (NM, CM and CD) were overexpressed and purified by immobilized affinity chromatography (IMAC). The purity was further polished by size exclusion chromatography (SEC). Zhang and colleagues have reported that dimers are virtually absent from CD mutant S100A4 proteins by electrophoretic analysis [24]. Our results showed that the WT, NM and CM were dimeric proteins and CD was a monomer protein in solution phase (Figure 5A). We also observed 95% purity of the purified S100A4 proteins from SEC by SDS-PAGE analyses (Figure 5B). To determine the secondary structural of mutants S100A4, the circular dichroism analysis was performed. Our results showed that NM and CM mutant S100A4 proteins increased the percentage of α-helix but CD mutant S100A4 protein reduced the content of α-helix. Additionally, NM S100A4 protein reduced the percentage of β-sheet, however, the CM and CD S100A4 proteins increased the content of β-sheet compared to the WT S100A4 (Figure 5C and 5D). Together, this data suggests that the C-terminal region of S100A4 is important for the maintenance of protein structure and dimerization.

### Summary of mutant S100A4 proteins on the CICs phenotypic properties

To demonstrate the effect of mutant S100A4 proteins on HN-CICs property, we first normalize the stemness phenotype of S100A4 WT and mutants in comparison with control (GFP) (Table 1). The cells harboring CM and CD S100A4 exhibited attenuated stemness phenotype compared to cells expressing WT S100A4. In addition, the cells harboring NM S100A4 were partially impaired the stemness phenotype in comparison with cells expressing WT S100A4. These results indicated that the C-terminal domain of S100A4 may be important for the maintenance of stemness properties of HN-CICs which might depend upon the protein structure of S100A4 (Figure 5). Furthermore, the cells expressing mutants S100A4 exhibited markedly reduced malignant phenotype in comparison with the cells harboring WT S100A4 (Table 2). These findings suggest that N-terminal and C-terminal region of S100A4 plays a distinct role of stemness and malignant properties of HN-CICs, in line with the idea that cancer stemness and EMT has distinct regulating mechanisms [43]. Collectively, the calcium-binding ability of S100A4 indeed sustains the stemness and malignant properties of HN-CICs.

**Table 1:** Stemness phenotype of S100A4 WT and mutants compared in the figures

Assay	WT	NM	CM	CD
OECM1 sphere formation	++++	+++	+	++
FaDu sphere formation	+++	++	±	+
^mem^GRP78-OECM1^f^	+++	++++	++	+
^mem^GRP78-FaDu^f^	++++	++	−	+++
GRP78 protein^W^	+++	±	±	−
Glut3 protein^W^	+++	+	+	+
Mice tumor volume	+++	−	+	++
CK18 (undifferentiation)^IHC^	+++	+	−	++
Nanog^IHC^	++++	++	+++	+
p-p53 ser15 (anti-apoptosis)^W^	+++	+++	−	−
Nanog^W^	++	±	−	−
Nanog reporter	+++	+	++	+
Total	38	18	4	9

Positive and negative determine as high and low expression levels in comparison with the GFP, respectively.

f flow cytometry

W immunoblotting assay

IHC immunochemistry

**Table 2:** Malignant phenotype of S100A4 WT and mutants compared in the figures

Assay	WT	NM	CM	CD
Migration-OECM1	+++	−	+	±
Migration-FaDu	++++	+	±	+
Anchorage independent growth-OECM1	++++	++	+	±
Anchorage independent growth-FaDu	++++	++	±	+
Vimentin^W^	++++	−	−	−
Total	18	2	0	1

Positive and negative determine as high and low expression levels in comparison with the GFP, respectively.

W immunoblotting assay

## DISCUSSION

According to the high incidence, HNSCC is still a major public health problem [44]. The major treatment modalities for HNSCC include surgery, chemotherapy and radiotherapy. Despite rapid advances in the diagnostic and surgical procedures, the survival rate of patients has not improved (about 45–50 %) [44–46]. Accumulating evidences show that there is a subpopulation of cancer cells, term as cancer-initiating cells (CICs), with the properties of chemoresistance and against irradiation treatment [8, 47]. Previously, we have demonstrated that the EMT mediator S100A4, a calcium binding protein, sustains the stemness properties of HN-CICs [26]. However, the S100A4-mediated mechanism that regulates HN-CIC properties remains elusive. In this study, our findings indicate that mutant S100A4 protein with loss-of-calcium-binding ability reduces the self-renewal properties of HN-CICs *in vitro* and *in vivo*.

S100A4 protein has been found to interact with many molecular targets that have been implicated in caner malignancy [23]. We have found that S100A4 knockdown causes significant changes of TP53 [26]. It has been reported that S100A4 inhibits the phosphorylation and promotes the degradation of p53 [32, 36]. In addition, we also found that the mutants S100A4 sphere cells are increased in the protein level of p-p53 ser15 (Figure 3C). Shen and colleagues report that resveratrol eliminates CSC properties by p-p53 ser15 activity [48]. As shown in Figure 3A and 3C, we found that the mRNA expressions of S100A4 is significantly and positively correlated with Nanog in both sphere cells and head and neck cancers, consistent with our prior finding that demonstrates the co-expression of S100A4 and Nanog in HNSCC patients [26]. In addition, p53 can directly bind to the promoter region of Nanog, resulting in suppressing its transcriptional activity [33]. In this study, we found that cell harboring mutants S100A4 decreased the protein level and promoter activity of Nanog (Figure 3D and 3E). These results suggest that S100A4 may role to inhibit p53 and subsequently activates Nanog to enhance the stemness properties.

In summary, we demonstrate that calcium-binding ability of S100A4 is involved in maintenance of stemness properties and tumorigenicity of HN-CICs. The mechanism is likely mediated via the inhibition of p53 that subsequently activates the Nanog transcriptional activity in Head and Neck cancers. Our study also reveals the possibility of targeting S100A4 as an effective modality to eliminate the HN-CICs.

## MATERIALS AND METHODS

### Cell lines cultivation and enrichment of HN-CICs from HNSCCs

Three HNSCC cell lines, SAS, FaDu and OECM1 [49], were grown in DMEM, MEM or in RPMI supplemented with 10% FBS (Grand Island, NY), respectively. For enrichment of HN-CICs, the three cell lines were cultured in tumor sphere medium consisting of serum-free DMEM/F12 medium (GIBCO), N2 supplement (GIBCO), 10 ng/mL human recombinant basic fibroblast growth factor-basic (FGF) and 10 ng/mL Epidermal Growth Factor (EGF) (R&D Systems, Minneapolis, MN). Cells were plated at a density of 7.5 × 10^4^ to 1× 10^5^ live cells/10-mm dishes, and the medium was changed every other day until the tumor sphere formation was observed in about 4 weeks [27].

### Construction of site directed mutagenesis

The protocol is provided by the QuikChange Lightning site-directed mutagenesis kit (Stratagene). The sequences of primer used for S100A4 E23A and D25A (NM), forward: 5′-CAACGCGGGTGCCAAGT TCAAGCTGAA-3′, reverse: 5′-GCACCCGCGTTGCCTG AGTATTTG-3′; for N65I, and D67A (CM), forward: 5′-AGCATCAGGGCCAATGAAGTTGAC-3′, reverse: 5′-GGCCCTGATGCTGTCCAAGTTG-3′. The N87Amber mutation (CD), forward 5′-TTGGCGCCTCGAGTAAT-3′, reverse: 5′-CAAAGAATTCCTAGCACATCATGGCA-3′ which designed by [24].

### Fluorescence-activated cell sorting (FACS) analysis

For cell surface marker identification, single cell suspension from trypsinized cells was stained with primary antibody anti-GRP78 (Miltenyi Biotec, Auburn, CA, USA) and secondary antibody conjugated with APC. Fluorescence intensity was detected by FACS Calibur apparatus (Becton Dickinson, San Diego, CA, USA).

### Immunoblotting

Protein extracts were prepared from the HNSCC or HN-CICs with RIPA buffer and protein content was measured with the protein assay kit (Bio-Rad). Thirty micrograms of proteins was boiled in sample buffer, separated by SDS/PAGE, and transferred onto nitrocellulose membrane. The membrane was incubated for 1 hr in blocking buffer [Tris-buffered saline with 0.1% Tween (TBS-T) and 5% nonfat dry milk] and incubated with primary antibodies (Supplementary Figure S5). After washing in TBS-T, the blot was incubated with horseradish peroxidase-conjugated secondary antibody, and the signals were visualized by the enhanced chemiluminescence system as described by the manufacturer (Perkin-Elmer, Wellesley, MA, USA). The blot was reprobed with *GAPDH* (Chemicon, Temecula, CA, USA) to confirm equal loading of the different samples.

### Subcutaneous xenografts in nude mice

All the animal practices in this study were in accordance with the institutional animal welfare guideline of National Yang-Ming University, Taiwan. HNSCCs or HN-CICs subject to treatment were injected subcutaneously into BALB/c nude mice (8 weeks). Tumor volume (TV) was calculated using the following formula: TV (mm^3^) = (Length × Width^2^)/2 and then analyzed using Image Pro-plus software.

### Microarray gene expression analysis

Gene profiling was measure by GeneSpring 12.6 using Affymetrix Human Genome U133 plus2.0. All sample files were preprocessed using “justRMA” and standardized as mean = 0 and SD = 1.

### *In vitro* cell migration assay

For transwell migration assays, 2 × 10^5^ cells were plated into the top chamber of a transwell (Corning, Acton, MA) with a porous membrane (8.0 μm pore size). Cells were plated in medium with lower serum (0.5% FBS), and medium supplemented with higher serum (10% FBS) was used as a chemoattractant in the lower chamber. The cells were incubated for 24 h at 37°C and cells that did not migrate through the pores were removed by a cotton swab. Cells on the lower surface of the membrane were stained with DAPI (Sigma-Aldrich) to show the nuclei; fluorescence was detected at a magnification of 100x using a fluorescence microscope (Carl Zeiss, Oberkochen, Germany). The number of fluorescent cells in a total of five randomly selected fields was counted.

### Anchorage independent growth assay

Each well (35 mm) of a six-well culture dish was coated with 2 ml bottom agar (Sigma-Aldrich) mixture (DMEM, 10% (v/v) FCS, 0.6% (w/v) agar). After the bottom layer was solidified, 2 ml top agar-medium mixture (DMEM, 10% (v/v) FCS, 0.3% (w/v) agar) containing 2×10^4^ cells were added, and the dishes were incubated at 37°C for 2 weeks. Plates were stained with 0.005% Crystal Violet then the colonies were counted. The number of total colonies with a diameter ≥100 μm was counted over five fields per well for a total of 15 fields in triplicate experiments.

### Protein overexpression and purification

The gene *S100A4* was cloned into a pET-21b vector between *NdeI* and *XhoI* restriction sites and transformed into *E. coli* BL21 (DE3). The recombinant wild-type S100A4, N-terminal Ca^2+^-binding site mutants E23A and D25A (E23A/D25A, NM), C-terminal Ca^2+^ binding site mutants N65I and D67A (N65I/D67A, CM) and C-terminal loop deletion S100A4 (designated as ΔS100A4, CD) were induced by adding 0.1 mM IPTG (isopropyl-β-D-thiogalactopyranoside) to the culture when cells reached an O.D._600_ of 0.6 and incubated at 37°C for 12hr. Cells were centrifuged at 8,000 rpm for 20 min, resuspended in buffer A (20 mM MES, 100 mM NaCl, 20 mM imidazole, pH 7.0) and disrupted by sonication. Supernatant was loaded into a Ni-NTA column (GE Healthcare) and unbound protein were washed away with 60 mM imidazole in buffer A. S100A4 proteins were eluted with 300 mM imidazole in buffer A. Fractions containing S100A4 proteins were dialyzed in buffer containing 10 mM CaCl_2_. Subsequently, S100A4 proteins were pooled and further purified by Superdex^TM^ 75 size exclusion chromatography (GE Healthcare) in buffer B (20 mM MES, 50 mM NaCl, 10 mM CaCl_2_, pH 7.0). Purified proteins were stored at 4°C for further use.

### Circular dichroism data collection and processing

The secondary structure of S100A4 was determined by circular dichroism spectroscopy. Circular dichroism spectra were measured on a Jasco J-715 spectropolarimeter at room temperature. Spectra were recorded between 185 and 260 nm, a 1.0-nm spectral step size, a 1.0-nm bandwidth, and a 20-nm/min scan rate using a 0.1-mm quartz cell. Six scans were averaged and corrected by subtracting six scans of the corresponding solvent to obtain the final spectra. The S100A4 and S100A4 mutants were concentrated to 3 mg/ml. BSA was used as control at the same concentration. The secondary structures of S100A4 were estimated by program K2D3, as described by Perez-Iratxeta et al. [50].

### Statistical analysis

Statistical Package of Social Sciences software (version 13.0) (SPSS, Inc., Chicago, IL) was used for statistical analysis. Student's *t* test or ANOVA was used to determine statistical significance of the differences between experimental groups; *p* values less than 0.05 were considered statistically significant. The level of statistical significance was set at 0.05 for all tests.

## SUPPLEMENTARY MATERIALS


