# Supplementary material for: Attenuation of cancer-initiating cells stemness properties by abrogating S100A4 calcium binding ability in head and neck cancers
Source: Oncotarget. 2016 Oct 26;7(48):78946–57. doi: 10.18632/oncotarget.12935 (PMC5346689; doi:10.18632/oncotarget.12935)
Supplement: Supplementary file 1 [file oncotarget-07-78946-s001.pdf]

Attenuation of cancer-initiating cells stemness properties by abrogating S100A4 calcium binding ability in head and neck cancers

Supplementary Materials

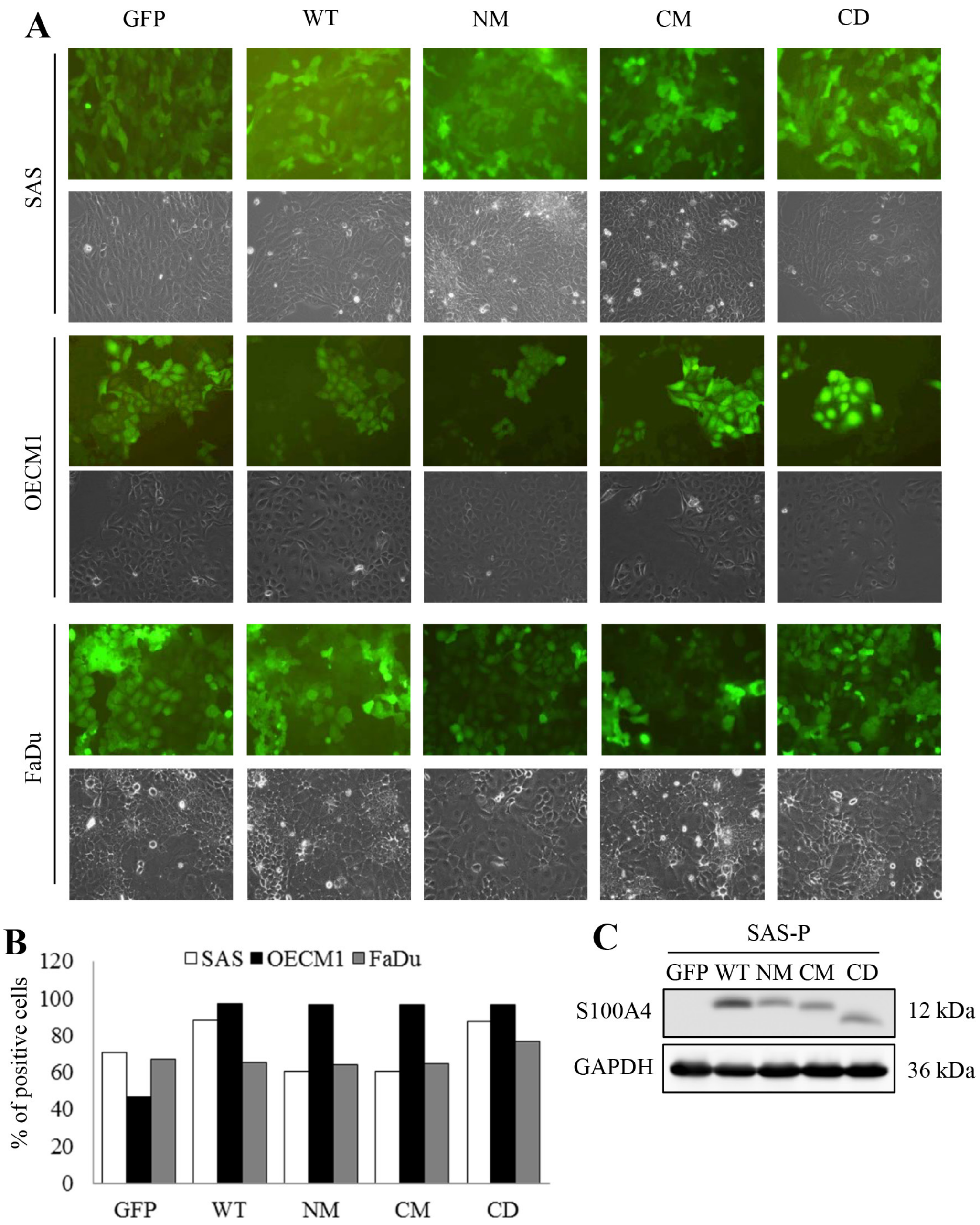

Supplementary Figure S1: Establishment of wild-type and mutants S100A4 overexpressing HNSCCs. (**A**) HNSCC cells, SAS, OECM1 and FaDu were infected with lentivirus co-expressing GFP and S100A4. (**B**) The percentage of GFP positive HNSCC cells after virus transduction. (**C**) Protein level of S100A4 and GAPDH in SAS-parental (P) was determined by immunoblot analyses.

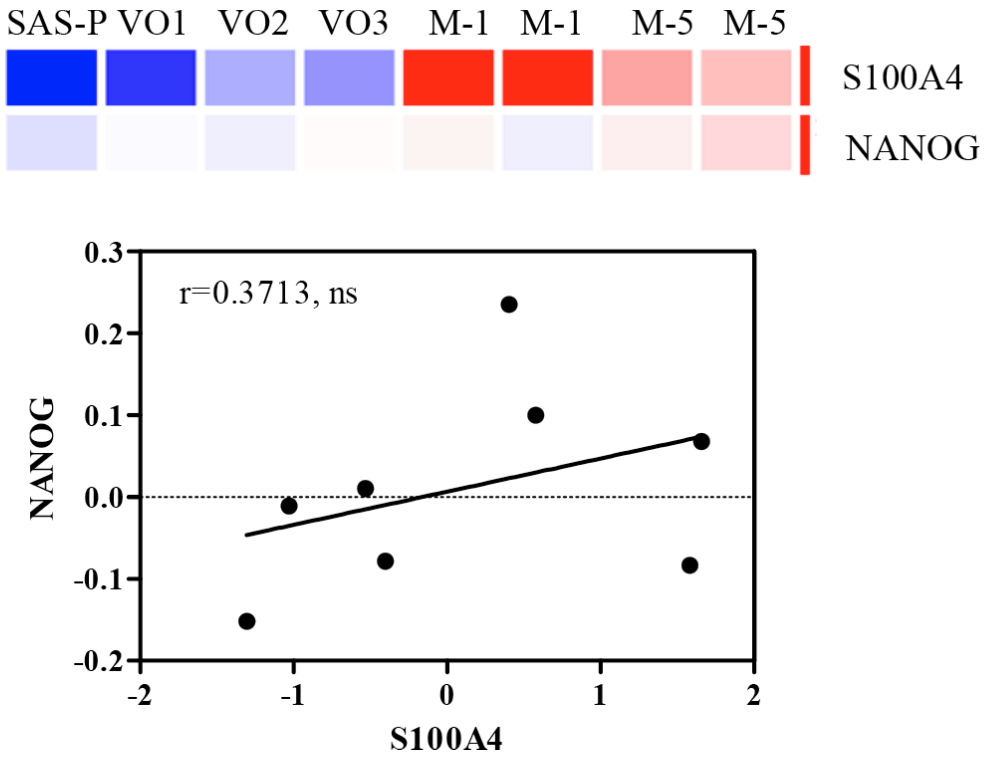

Supplementary Figure S2: The gene correlation of S100A4 and Nanog of SAS derived *in vivo* cell lines. The heat maps of the S100A4 and Nanog of SAS derived in vivo cell lines. Red and blue determine as high and low expression levels, respectively (top panel). Expression correlation analysis of S100A4 (X-axis) and Nanog (Y-axis) (bottom panel).

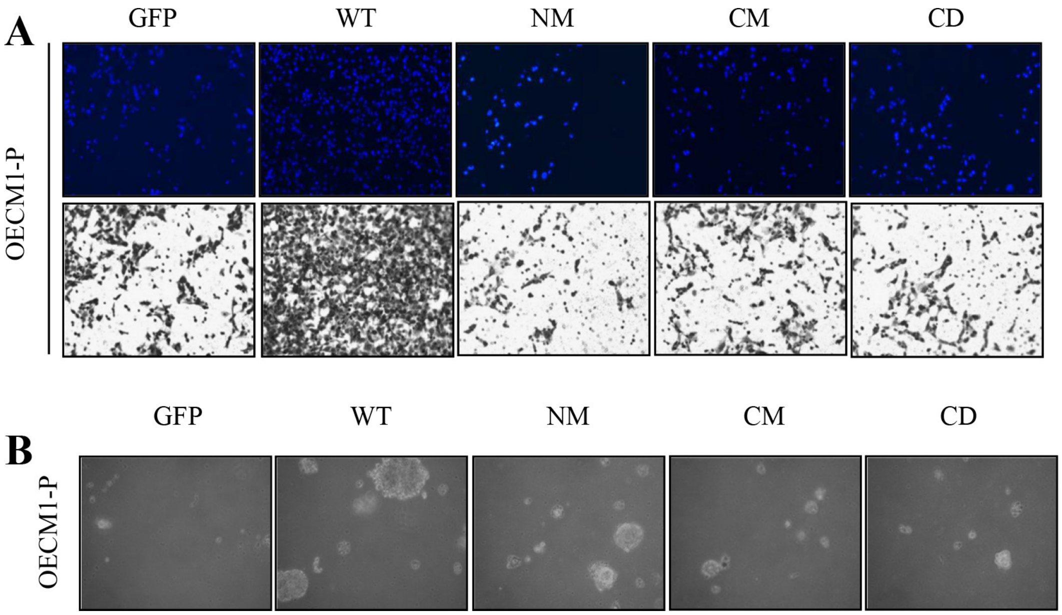

Supplementary Figure S3: The mutants S100A4 reduce the tumorigenic capacity of OECM1 cells. (**A**) The migration ability of OECM1-P cell lines was examined as described in materials and methods. (**B**) Anchorage-independent growth of S100A4-overexpressing OECM1-P cell lines was analyzed.

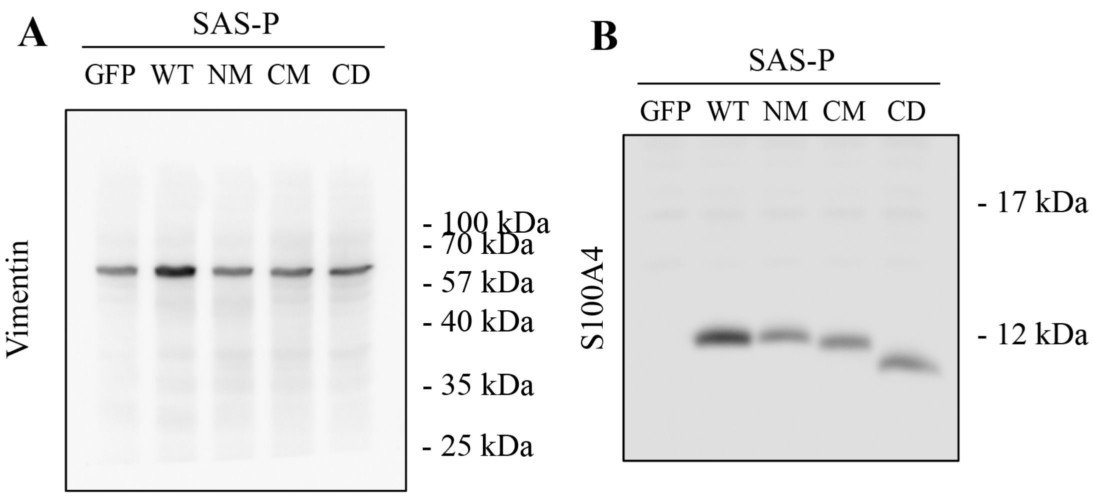

Supplementary Figure S4: The specificity data of antibodies. The immunoblotting analyses of Vimentin (**A**) and S100A4 (**B**) were showed. The proper molecular markers were labeled.

A

**Primary antibodies**	**Catalog number**	**Commercial company**	**Reference**
Actin	MA5-15739	ThermoFisher	[1, 2]
CK18	#4548	Cell Signaling Technology	[3, 4]
E-cadherin	610182	BD Biosciences	[5-7]
GAPDH	MAB374	EMD Millipore	[8, 9]
Glut3	ab15311	abcam	[10-12]
GRP78	610978	BD Biosciences	[13-15]
Nanog	AB9220	EMD Millipore	[16-18]
p-p53 ser15	#9284	Cell Signaling Technology	[19, 20]
p53	#9282	Cell Signaling Technology	[21, 22]
S100A4	A5114	Dako	[23, 24]
Vimentin	550513	BD Biosciences	[23, 25]

B Reference

1. Zhang YM, Dai QF, Chen WH, Jiang ST, Chen SX, Zhang YJ, Tang CZ and Cheng SB. Effects of acupuncture on cortical expression of Wnt3a, beta-catenin and Sox2 in a rat model of traumatic brain injury. Acupunct Med. 2016; 34(1):48-54.
2. Loffler MG, Birkenfeld AL, Philbrick KM, Belman JP, Habtemichael EN, Booth CJ, Castorena CM, Choi CS, Jornayvaz FR, Gassaway BM, Lee HY, Cartee GD, Philbrick W, Shulman GI, Samuel VT and Bogan JS. Enhanced fasting glucose turnover in mice with disrupted action of TUG protein in skeletal muscle. The Journal of biological chemistry. 2013; 288(28):20135-20150.
3. Wang X, Lao Y, Xu N, Xi Z, Wu M, Wang H, Li X, Tan H, Sun M and Xu H. Oblongifolin C inhibits metastasis by up-regulating keratin 18 and tubulins. Scientific reports. 2015; 5:10293.
4. Yin G, Alvero AB, Craveiro V, Holmberg JC, Fu HH, Montagna MK, Yang Y, Chefetz-Menaker I, Nuti S, Rossi M, Silasi DA, Rutherford T and Mor G. Constitutive proteasomal degradation of TWIST-1 in epithelial-ovarian cancer stem cells impacts differentiation and metastatic potential. Oncogene. 2013; 32(1):39-49.
5. Wu CH, Tang SC, Wang PH, Lee H and Ko JL. Nickel-induced epithelial-mesenchymal transition by reactive oxygen species generation and E-cadherin promoter hypermethylation. The Journal of biological chemistry. 2012; 287(30):25292-25302.
6. Shen L, Qin K, Wang D, Zhang Y, Bai N, Yang S, Luo Y, Xiang R and Tan X. Overexpression of Oct4 suppresses the metastatic potential of breast cancer cells via Rnd1 downregulation. Biochimica et biophysica acta. 2014; 1842(11):2087-2095.
7. Cole BK, Curto M, Chan AW and McClatchey AI. Localization to the cortical cytoskeleton is necessary for Nf2/merlin-dependent epidermal growth factor receptor silencing. Molecular and cellular biology. 2008; 28(4):1274-1284.
8. Bach C, Gilch S, Rost R, Greenwood AD, Horsch M, Hajj GN, Brodesser S, Facius A, Schadler S, Sandhoff K, Beckers J, Leib-Mosch C, Schatzl HM and Vorberg I. Prion-induced activation of cholesterogenic gene expression by Srebp2 in neuronal cells. The Journal of biological chemistry. 2009; 284(45):31260-31269.
9. Gold JI, Martini JS, Hullmann J, Gao E, Chuprun JK, Lee L, Tilley DG, Rabinowitz JE, Bossuyt J, Bers DM and Koch WJ. Nuclear translocation of cardiac G protein-Coupled Receptor kinase 5 downstream of select Gq-activating hypertrophic ligands is a calmodulin-dependent process. PloS one. 2013; 8(3):e57324.
10. Kaira K, Oriuchi N, Takahashi T, Nakagawa K, Ohde Y, Okumura T, Murakami H, Shukuya T, Kenmotsu H, Naito T, Kanai Y, Endo M, Kondo H, Nakajima T and Yamamoto N. L-type amino acid transporter 1 (LAT1) expression in malignant pleural mesothelioma. Anticancer research. 2011; 31(12):4075-4082.
11. Kaira K, Ohde Y, Endo M, Nakagawa K, Okumura T, Takahashi T, Murakami H, Tsuya A, Nakamura Y, Naito T, Kondo H, Nakajima T and Yamamoto N. Expression of 4F2hc (CD98) in pulmonary neuroendocrine tumors. Oncology reports. 2011; 26(4):931-937.
12. Mees G, Vangestel C, Dierckx R, Loomans S, Van Damme N, Peremans K, De Rooster H, Van Goethem B, Pauwels P, Ducatelle R and Van de Wiele C. Metabolic correlates of tumour hypoxia in malignant canine mammary carcinoma. Res Vet Sci. 2011; 91(3):e125-128.
13. Morishima N, Nakanishi K, Takenouchi H, Shibata T and Yasuhiko Y. An endoplasmic reticulum stress-specific caspase cascade in apoptosis. Cytochrome c-independent activation of caspase-9 by caspase-12. The Journal of biological chemistry. 2002; 277(37):34287-34294.
14. Yamazaki T, Chang TY, Haass C and Ihara Y. Accumulation and aggregation of amyloid beta-protein in late endosomes of Niemann-pick type C cells. The Journal of biological chemistry. 2001; 276(6):4454-4460.
15. Wu MJ, Jan CI, Tsay YG, Yu YH, Huang CY, Lin SC, Liu CJ, Chen YS, Lo JF and Yu CC. Elimination of head and neck cancer initiating cells through targeting glucose regulated protein78 signaling. Molecular cancer. 2010; 9:283.
16. Tan G, Cheng L, Chen T, Yu L and Tan Y. Foxm1 mediates LIF/Stat3-dependent self-renewal in mouse embryonic stem cells and is essential for the generation of induced pluripotent stem cells. PloS one. 2014; 9(4):e92304.
17. Chang CW, Chen CC, Wu MJ, Chen YS, Chen CC, Sheu SJ, Lin TW, Chou SH, Lin SC, Liu CJ, Lee TC, Huang CY and Lo JF. Active Component of Antrodia cinnamomea Mycelia Targeting Head and Neck Cancer Initiating Cells through Exaggerated Autophagic Cell Death. Evidence-based complementary and alternative medicine. 2013; 2013:946451.
18. Tsai LL, Yu CC, Chang YC, Yu CH and Chou MY. Markedly increased Oct4 and Nanog expression correlates with cisplatin resistance in oral squamous cell carcinoma. Journal of oral pathology & medicine. 2011; 40(8):621-628.
19. Lee WJ, Jo SY, Lee MH, Won CH, Lee MW, Choi JH and Chang SE. The Effect of MCP-1/CCR2 on the Proliferation and Senescence of Epidermal Constituent Cells in Solar Lentigo. International journal of molecular sciences. 2016; 17(6).
20. He D, Xiang J, Li B and Liu H. The dynamic behavior of Ect2 in response to DNA damage. Scientific reports. 2016; 6:24504.
21. Wang J, Hu K, Guo J, Cheng F, Lv J, Jiang W, Lu W, Liu J, Pang X and Liu M. Suppression of KRas-mutant cancer through the combined inhibition of KRAS with PLK1 and ROCK. Nature communications. 2016; 7:11363.
22. Fan L, Peng G, Sahgal N, Fazli L, Gleave M, Zhang Y, Hussain A and Qi J. Regulation of c-Myc expression by the histone demethylase JMJD1A is essential for prostate cancer cell growth and survival. Oncogene. 2016; 35(19):2441-2452.
23. Lo JF, Yu CC, Chiou SH, Huang CY, Jan CI, Lin SC, Liu CJ, Hu WY and Yu YH. The epithelial-mesenchymal transition mediator S100A4 maintains cancer-initiating cells in head and neck cancers. Cancer research. 2011; 71(5):1912-1923.
24. Orre LM, Panizza E, Kaminskyy VO, Vernet E, Graslund T, Zhivotovsky B and Lehtio J. S100A4 interacts with p53 in the nucleus and promotes p53 degradation. Oncogene. 2013; 32(49):5531-5540.
25. Chen YS, Wu MJ, Huang CY, Lin SC, Chuang TH, Yu CC and Lo JF. CD133/Src axis mediates tumor initiating property and epithelial-mesenchymal transition of head and neck cancer. PloS one. 2011; 6(11):e28053.

Supplementary Figure S5: The antibodies list. The list of the primary antibodies used in the study (**A**) and references of primary antibodies (**B**) were showed.
